# Supplementary material for: Observations of marine animal interactions with a small tidal turbine
Source: PLoS One. 2026 Jan 14;21(1):e0338376. doi: 10.1371/journal.pone.0338376 (PMC12803451; doi:10.1371/journal.pone.0338376)
Supplement: S4 Detailed Description of Human Review Process — Review Process.pdf provides a more detailed overview of the human review process for optical images described in the manuscript. (PDF) [file pone.0338376.s004.pdf]

Overview of the human review process

The process of reviewing and annotating acquired optical images, performing taxonomic classification, determining interaction types, and exporting relevant videos was carried out a group of reviewers as summarized in Figure S1. This process was broken into several steps involving initial reviewers (R_i) and a single, final reviewer (R_f). First, the initial reviewers (x4) and final reviewer viewed individual optical images captured by the AMP. Every image contained in the three data subsets was reviewed by at least one initial reviewer. Optical images were reviewed one at a time to make it easier for reviewers to spend more time observing individual frames and zoom in on portion of the images to address any ambiguities. Prior to beginning their reviews, the initial reviewers were briefed by the final reviewer and shown example images of seabirds, seals, and fish. Initial reviewers were told to flag any frame in which a target of interest was positively identified as well as those where observations were ambiguous (e.g., floating plant matter had the shape or a bird or a small fish). Initial reviewers maintained a log of events (i.e., the timestamps of the first and last frames in which the animal or potential animal was observed), which included an initial classification (bird, fish, seal, or unknown).

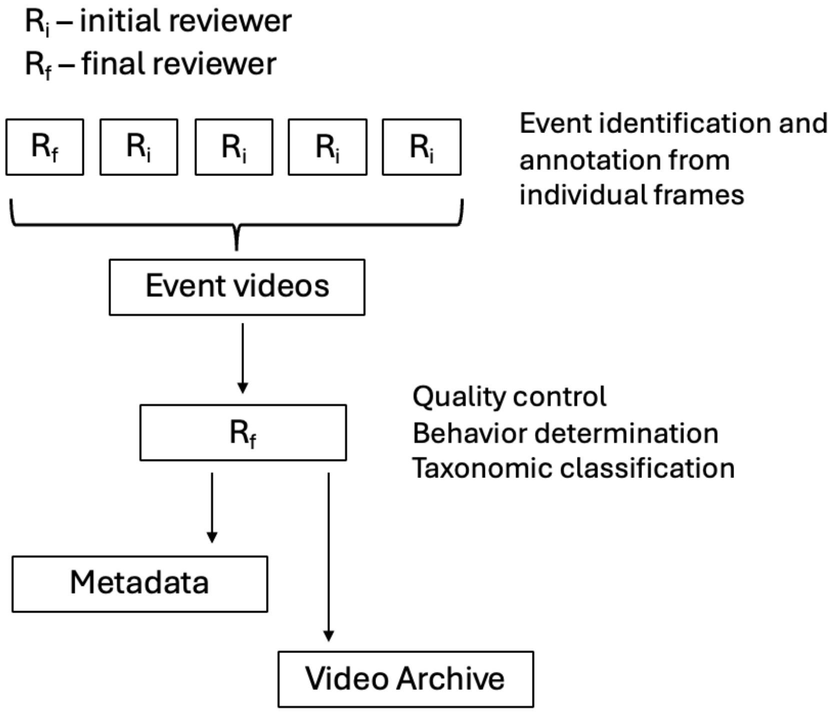

Figure S1. An overview of the manual review process for all optical images acquired during in all three data subsets.

Once initial review was complete, all frames corresponding to individually annotated events were exported to videos for quality control and final classification by the final

reviewer. This final review aimed to ensure that identified events were consistent across the reviewers and that events had appropriate taxonomic classifications. If necessary, individual frames were revisited and event timestamps were modified. Behavioral classifications (e.g., evasion, collision) were also made by the final reviewer.

Because initial reviewers were encouraged to identify ambiguous objects in the imagery, the final reviewer removed many events that were ultimately not classified as fish, seabirds, or seals. The total number of events rejected by the final reviewer was not tracked but was small for seabirds and seals (i.e., these animals were typically unambiguous and readily identified). However, in the case of small fish, more potential targets were removed from the data set than were ultimately retained for further analysis. These targets were removed if they could not unambiguously be determined to be small fish (i.e., they showed clear physiological features of fish or were observed swimming). Thus, small fish passive drifting passively through the frames may have been removed from the final event counts. *We therefore believe that small fish were likely significantly undercounted, but the final event counts include all fish that were clearly maneuvering around the turbine.*

To ensure that initial reviewers were consistently and reliably annotating potential animal interactions, in several cases, the same period of data was reviewed by two initial reviewers. This was performed when reviews of the metadata compiled by the final reviewer suggested potential biases in review (e.g., seabirds were regularly seen in data annotated by one initial reviewer while other initial reviewer had few similar annotations under similar conditions just days earlier or later). These secondary reviews did not capture any additional events missed by the initial reviewers following quality control by the final reviewer. We attribute this to two factors. First, most events associated with large fish, seabirds, and seals are easily identified. Second, differences in the annotations from initial reviewers were often associated with ambiguous events that were ultimately removed in quality control.

The most common uncertainties identified in the quality control review process varied by taxonomic classification. Uncertainties for each animal group are described below.

Seals: Mischaracterization, or uncertainty, regarding potential seal events was limited to shadows on the edges of the frame or in the distance behind the rotor. When seal-like motion or visual characteristics of seals could not be identified, the events were discarded. This meant that some events where a seal was in the vicinity of the turbine may have been discarded, but we did not over-count seals, and would not have discarded any events where seals approached the turbine.

Seabirds: In a surprisingly large number of cases, drifting plant matter (kelp) had shapes that looked like a diving seabird in an individual frame. However, this plant matter drifted passively through the frame making it relatively easy to identify in videos compared to individual optical frames. While some instances of kelp were identified as potential seabirds by initial reviewers, determination by the final reviewer was largely unambiguous.

Fish: The most common source of uncertainty in the classification of small fish events was small bits of floating plant matter that were present in the water column throughout the deployment. Ambiguities were most common during low-light periods when strobe lights were on, because the light reflected off both fish and plant matter. Figure 6a in the manuscript provides a good example of a small fish illuminated near the camera that was easy to identify due to its motion and defining features. However, had the target been located further from the cameras or been drifting passively through the frames, it could not have been positively identified without a high degree of uncertainty. Plant matter is visible in the background of several of the frames in Figure 6.
