# Supplementary material for: Observations of marine animal interactions with a small tidal turbine
Source: PLoS One. 2026 Jan 14;21(1):e0338376. doi: 10.1371/journal.pone.0338376 (PMC12803451; doi:10.1371/journal.pone.0338376)
Supplement: Attachment — Submitted filename: PlosONE Response to Reviewers.pdf [file pone.0338376.s006.pdf]

Dear Associate Editor and Reviewers,

First, we want to express our appreciation for the efforts made by the reviewers. We have reviewed their comments in detail, have made all requested changes (some with modest modifications), and believe these revisions have improved the manuscript. We have also added a reference to a recently published review paper (Ascher 2025) and made minor edits for clarity throughout. We appreciate some of the additional commentary provided by the reviewers regarding the challenges of this sampling and the general importance of the lessons learned. We share a similar view and hope that was well reflected in the manuscript. Below you will find our responses to all of the requests for revisions. Initial reviewer responses are in black while author responses are in red, and a redline version of the manuscript is attached. We hope that all parties find that with these revisions the manuscript is suitable for publication in PlosONE. Thank you again for taking the time to provide a thoughtful review of our work.

Author responses

3. Have the authors made all data underlying the findings in their manuscript fully available?

Reviewer #1: No

Reviewer #2: No

The data that underlie Figures 4 and 5 and Table 2 are included in the supplemental data. All videos that are described in the text and include the stills contained in Figures 6-11 were uploaded to the repository but were not yet live at the time of submission. As of 1 October 2025 the data are confirmed to be available and have been downloaded several times. This should resolve comments made by both reviewers in response to the availability of the data.

5. Review Comments to the Author

Reviewer #1: No -for date. There is a repository (see detailed notes below) listed in the paper, but data don't seem to have been uploaded yet. Once this is resolved, I'm happy that the answer to Q3 above will be yes.

See note above in response to 3 (i.e., data are now available on the repository).

Other comments

Reviewer 1
A welcome paper both providing useful insights into technologies for monitoring animal movement around tidal energy systems and also highlighting some of the difficulties in detecting small animals close to large bits of moving machinery. Some of the conclusions are a not very conclusive, but that is the nature of these data and I think it's important that it's published, in part as a statement of how difficult this type of work is, and to inform people that there is not (anytime soon at least) going to be a magical system that can process these types of data automatically (i.e. cheaply) as the tidal energy industry expands.

We appreciate this feedback and will readily admit our own hesitation around this work given the conclusions “are not very conclusive.” None the less, we see the same value as the reviewer in providing these perspectives. Thank you for sharing your views on the subject.

Introduction

L 57: “harbor seal presence” This study could not tell the difference between harbor and grey seals, both present in the area, so it’s just “seal presence”, or “harbor and grey seal presence”.

Thank you for this correction; the word “harbor” has been removed.

L76. What do the terms meso and macro mean ?. So far as I can tell, meso scale avoidance is the same as the definition of avoidance that you took earlier from Garavelli, and micro avoidance is what you’ve earlier called evasion. I think you should stick to one set of terms. Avoidance and Evasion were quite nicely defined earlier, so maybe best to stick with them, so this might read: “Further, most studies to date have studied animal avoidance at larger scales, but there have been few reports of fine scale evasion behavior in the immediate vicinity of a tidal turbine”

This is a good suggestion, and the original language was unclear. We have revised per the reviewer’s suggestion to use the avoidance/evasion terminology. The original text stated:

> “Further, most studies to date have studied animal avoidance at the meso- or macro-scale, but there have been few reports of micro-scale animal behavior in the immediate vicinity of a tidal turbine.”

It now states:

> “Further, most studies to date have investigated animal avoidance at larger scales, but there have been few reports of fine-scale evasion behavior in the immediate vicinity of a tidal turbine.”

Methods

L94: ‘in several’ or ‘on several’ ?

This has been revised to “on several”.

L112 – 115: Should this be in a separate section on Ethics and Permits ?

Both reviewers made comments related to this. We agree and have removed the following statement from the aforementioned lines and moved it to the Ethics statement.

“Deployment of the Turbine Lander proceeded after receiving a categorical exclusion from the Department of the Navy following review of the proposed project by several state and federal agencies, including the National Marine Fisheries Service and U.S. Fish and Wildlife Service. Sampling was limited to optical and acoustic imagery; no animals were directly sampled.”

Around L137: Can you say more about how the fields of view of the sonars were organised. From figure 1, it looks as though the two BlueView's have the same vertical orientation, but are about 90 deg separate horizontally, giving a combined swath of over 180 degrees ? Why didn't you orientate them the same horizontally and at different vertical angles to cover the full height of the device ? Also, did you sample these at the same time as the Gemini ? Was there interference – although they are at different frequencies, I'm guessing quite bad side lobes ?

We have added the following text to the manuscript in response to this comment:

> "To facilitate imaging of animals both up and downstream of the turbine during both flood and ebb tides, the BlueViews were oriented perpendicular to each other such that their beams overlapped by approximately 45° at the rotor. The Gemini was oriented such that the rotor was centered in its field of view. The AMP software triggered acquisition of the acoustic images with short time delays (e.g., < 50 ms) to avoid crosstalk while minimizing elapsed time between images."

Additionally, we have added a reference to our recent conference publication that provides more details on the acoustic camera data: "A detailed description of the acoustic camera data can be found in Bassett and Cotter [26]" is now included at the end of this section.

More information:

The reviewer is correct, the BlueViews were oriented such that their fields of view overlapped by approximately 45 degrees at the same height above the seabed. The sonars were controlled by the AMP to ping at slightly different times to avoid crosstalk. As with the optical sampling, modifications to timing of the sonars were made during the deployment. Representative offsets in timing between the sonars was on the order of 10 to 30 ms, which still allows for 10 Hz ping rates on all sensors with minimal cross talk. A typical approach was to acquire images in the following order: BlueView 1, Gemini , BlueView 2, with timing offsets of 0 ms, 25 ms, 50 ms (relative to BlueView 1). Due to the higher frequency of the Blueviews, there is some potential for crosstalk between Blueview 2 and the Gemini or the Gemini and Blueview 1, but iterative testing suggested this wasn't a significant issue with this timing.

The intent of this design was to capture animals upstream of the turbine during both flood and ebb tides. In practice, the close range of the turbine (<2 m) meant that only a slice of the turbine was ensonified, which limited the utility of the sonar data. That said, we recently published a conference paper in the Proceedings of the European Wave and Tidal Energy Conference focused on the sonar data and corresponding lessons learned. Because the focus of this manuscript is on the optical camera paper, and the methods are already fairly long, we have not included all of these details in this paper. However, we have added a reference to the conference proceeding for readers interested in the subject. We also note that sonar data from dozens of the optical events in this paper have been uploaded to the same publicly available dataset.

Vertical orientation of the sonars will certainly be considered in future deployments of this system, and we also note that we anticipate placing sensors further from the system to capture the full rotor in future work.

L160 or earlier at around 134: can be more explicit about when you operated the lights ? There is some information at L165, and again at line 175, but was the strategy ? Were they only used after model detections ?

We agree that this detail was missing and have added text describing strobe light activation during scheduled data acquisition: strobe lights were activated during duty cycle windows, but not during continuous 1 Hz data acquisition. Strobe lights were always activated for 2.5 s after model detections.

L168 '… using their …' is this referring to MarineSitu or Ultralytics ? Do you mean 'deployment' software, or 'detection' software ?

MarineSitu software was used; this has been clarified.

At L171 you say the model reliably detected seals. At L180 and again at line 185 you say that it did not perform well for that it performed poorly. There is a contradiction here that needs to be resolved.
We see the potential contradiction here and, having reviewed the text, feel that the relevant statements reflect our intended meaning. Specifically, the first comment relates to the model operating on optical camera data, while the second is related to the model operating on the acoustic camera data. Thus, we have not modified this text.

On what was line 171 in the initial draft the manuscript states "[t]he optical camera detection model was trained to detect seals, diving seabirds, and fish, however it only reliably detected seals and diving seabirds,..."

The stated contradiction on what was line 180 states "[a]coustic camera detection models (only deployed after 1 February) were trained to detect seals and diving birds, but did not perform well."

L182: At this point, you start to bring results into the methods section. While I can see that you do need some generalities about the results here, to give context to the iterative methods, try to keep specific results as much as possible to the Results section.

Our intent was to keep the description of the models in the methods, because they were a tool used for data collection, and focus the results on the acquired dataset. We chose to keep these details in the methods because model performance was not rigorously assessed, particularly for the acoustic camera detection models.

L306: The video repository Basset & Cotter, https://mhkdr.openei.org/submissions/599 appears to still be in progress and doesn't yet have any videos available.

Please refer to comments near the top of our response as this has been resolved.

Discussion

Fish collision rate. It seems that you rate of collision is still about 2%, which is not entirely insignificant. I know that for marine mammals, regulators are currently using a range of evasion / avoidance values, though 98% seems to be one that's popular. Are there any similar numbers for fish, that are currently being used in collision risk models, that you could compare your numbers to ? I'm guessing that you're staying away from doing this because you think the 4 collisions is accurate, but the 229 fish is a vast underestimate.

While there has been some effort to quantify the probability of fish collisions (e.g., Peraza and Horne 2023[1]), to our knowledge, there are not probability values that are routinely used by regulators (at least in the United States). We are hesitant to relate our results to rates or probabilities of collision/avoidance for three reasons. First, as you mentioned, we believe that the 229 fish that we identified around the moving turbine is a significant underestimate due to the difficulty in discerning small fish, particularly during the periods with the strongest currents. We have added statements to the abstract and conclusions to further emphasize this point. Second, it is likely that animals avoided the turbine outside of the optical camera field of view; so our observations would be limited to evasion events. Finally, because of the nature of our sampling, we do not feel that we should draw statistical conclusions from the data.

That said, in the discussion, we do emphasize that the observed collisions represent a small fraction of recorded fish encounters with the turbine. We have left the text regarding this topic as it was in the original submission, but hope to better address this question in future studies with the same turbine and monitoring system. We have also emphasized that fish were likely undercounted throughout the paper (abstract, conclusions).

L466. You state here that the real time detection model was not trained to detect fish. I thought that one of the main things you'd been doing was retraining and redeploying a model on the early data to specifically detect fish. This sentence seems to be in direct contradiction of the methods.
Thank you for pointing out this inconsistency. The language in the methods is correct; the model was trained with annotations of fish, birds, and seals, but did not perform well for fish. For large fish, this was due to the limited training data available (detections were rare). For small fish, this was due to the difficulty in discerning small fish (for both humans and machines).

The statement in the discussion section has been revised:

---

[1] Peraza, J. I. and Horne, J. K. 2023. Quantifying conditional probabilities of fish-turbine encounters and impacts. *Frontiers in Marine Science*. doi: 10.3389/fmars.2023.1270428

“…the real-time detection model that triggered data collection at a sufficiently high frame rate to resolve fine-scale fish behavior did not effectively detect fish.”

Line 484: Can you clarify this please. You making a comparison between a speed and a length, which is not really possible.

We agree that this sentence was poorly worded, and have rephrased and added additional context to clarify the point. For reference, the initial text stated:

> “For these small fish, flow speeds typically exceeded the length of the fish by more than a factor of 10. Under these conditions, many fish can only swim for short periods of time against the currents.”

This is poorly worded as the more appropriate phrasing would have been wordy, but something like “For these small fish, during turbine operation flow speeds typically exceed a value of 10 body lengths per second. Therefore, burst swimming speeds, in conjunction with the speed of the moving blades, could be used to evaluate the ability of fish to evade moving turbine blades.”

This is important as there is considerable evidence in the literature that swimming speeds on the order of 10 body lengths per second are at the upper limit of what most species can achieve even for short periods of time while maintaining speeds for long periods requires significantly lower values. Further literature reviews continue to build on the cited works. We think this is an important thing to point out as it clearly relates to collision risk. Furthermore, it’s possible that swimming capabilities explain why we did not observe large fish near the turbine during operation. However, that last point is strictly speculative but has been added to the paper with that caveat. We have edited the statement in the paper to state the following:

> “For these small fish, flow speeds during turbine operation typically exceed a value of 10 body lengths per second. This threshold corresponds to the upper limit of swimming speeds that most fish can achieve for short periods of time (burst swimming), while sustained swimming speed thresholds would be expected to be lower (Bainbridge 1958, Sambilay 1990). We hypothesize that the inability of small fish to swim against the current in a sustained manner may explain why most fish observed during turbine operation were small (i.e., large fish with stronger swimming capabilities may have avoided the turbine outside of the camera field of view). If true, burst swimming speeds, in conjunction with the speed of a turbine's blades, could be used to evaluate fish evasion capabilities.”

Lines 491 to 494. Can you clarify whether the difficulty is with AI models, human observers, or both please. Do you have numbers on how many ambiguous targets there were as well as the 229 positive identifications. I think that quantifying the uncertainty in all these measurements is important in guiding how future studies might be implemented and what level of data they might provide to regulators and other interested parties. I think you need to expand the results section

a bit
Distinguishing between fish and plant matter was quite difficult (in many cases, arguably impossible) for human reviewers. This has been clarified in the text:

> "Distinguishing between drifting plant matter and small (e.g., less than the foil chord length; 10 cm) fish in the optical data was difficult *for human reviewers*…."

We also have added additional supplemental information that discusses the review process. The initial reviewers were asked to identify all potential, but ambiguous targets and the final reviewer performing quality control decided which targets to include. A decision was made to remove the ambiguous targets. While a subjective decision, this was preferred over inflating target numbers. While not tracked, it would be reasonable to estimate that approximately an order of magnitude more potential small targets of interest were removed due to ambiguity. While perhaps a mistake, in retrospect, the decision not to track these was made early on in our analysis simply due to the volume of data and short project timelines.

L548 soft start – or use of a deterrence device for a few seconds prior to startup ?

This is a good suggestion, we have added this to the text:

"Alternatively, a deterrence device could be activated for a short period before turbine start-up."

L558: I'm pretty sure the tip speed of the turbines in the Pentland Firth is around 13m/s (9m radius, 14rpm), in a 3-4m/s current.

Thank you for the comment. This is correct and the number in the initial draft was site driven. Comparable tip speed ratios are, of course, typical for axial-flow turbines at larger scales and therefore higher speeds would be expected. We have not edited our text since this was intended to specifically point out what would be expected at the same site.

L562. Do you know the mass of the blades in your system ? For the big turbines, it's so much bigger than the mass of an animal that the animal is going to get a hard whack and barely slow the blades at all. For your turbine it looks as though the blades would probably (or have?) come of worse in a collision because they are probably lighter than a seal. Probably too much of a rabbit hole to discuss this, but you might put the mass of the system into the introduction somewhere so that people can do their own math on this if they are that way inclined.
The mass of the blades in air was 2.68 kg. Its wet weight is, of course, a bit less. We note, however, that the moment of inertia is the most important term for assessing the potential impacts if a collision were to occur. We have edited the manuscript to include the foil wet weight and moment of inertia. The Methods previously stated:

> "While small (the four-bladed rotor is 1.19 m tall, 0.85 m in diameter), the system is considered full-scale as the target application is to provide modest amounts of power at sea where no cabled infrastructure exists."

It now states:

> "The four-bladed rotor is 1.19 m tall and 0.85 m in diameter with blade chord lengths of 10.2 cm. The individual blades have a wet weight of approximately 1.5 kg and the turbine's moment of inertia is estimated to be approximately 2 kg m^2. When deployed, the bottom and top of the rotor are approximately 1.5 and 2.7 m above the seabed, respectively. While small, the system is considered full-scale as the target application is to provide modest amounts of power at sea where no cabled infrastructure exists."

L583: I think you could remove "particularly for fish", or say "for small or animals at any range, and even for larger animals at greater ranges".

We have revised this sentence accordingly, and removed the text about "species identification" to further clarify this statement, because species identification is rarely possible with acoustic cameras unless there is only one species with a particular morphology present. The first version stated:

> "but their low resolution and frame rate (depending on range requirements) limit species identification and behavior classification capabilities, particularly for fish."

and now says

> "...but their low resolution and frame rates (depending on range requirements) limit behavior classification capabilities for small animals, and even for larger animals at greater ranges."

L628: 175GByte per day. Hard yes, but can be done, particularly at this pilot stage of developing these systems.

We agree - this is certainly doable, but, as we have stated in the text, unwieldy.

L660: "Once the model achieves …". What if they don't ? Everyone seems to be taking that attitude that the AI will eventually be good enough. Even in your own training data, you say you've a lot of targets that you don't know what they are – fish or debris. How do you even handle them in model training ? While I agree that with time, you'll make better algorithms, and you'll be able to train them with more data, I don't actually believe they will be that much better than the current ones that are inadequate in many ways. Perhaps you need to stress in these final paragraphs the importance of keeping a human in the loop, and of having review software that can allow the human to efficiently examine these events. Getting that software right might be more beneficial that continually bang

We agree that model performance in the dynamic environments where tidal turbines are deployed is a challenge that is not going to go away, and that we will likely never avoid having a "human in the loop". However, we believe that it is not financially viable, and poses administrative challenges, to continue to acquire and analyze the volume of data needed to address questions about collision risk without the use of automatic detection and classification

algorithms. That said, our dataset highlights the challenges in doing this, particularly for small fish. We have revised this paragraph as follows:

- Changed “Once the model achieves…” to “If the model achieves…”
- We have also added some text (beginning ~L682) about the possibility of model failure:

  “Depending on the species of interest and the image quality, in some cases, suitable model performance may not be achievable. For example, reliable detection of small fish was challenging for human reviewers in our dataset, and it is unlikely that a machine learning algorithm will be able to detect them reliably. In this case, duty cycles or continuous data acquisition are recommended.”

Reviewer #2

Some more comments below and in the attached pdf.

We have inserted a section below dedicated to highlighting modifications made in response to the annotated pdf.

Authors have put N/A under ethics statement which also includes field work. Although the work does not involve collection of samples, surely there would have been permissions/permitting process to install the tidal turbine and associated instrumentation for the work.

This is a valid point. We have modified the ethics statement to note that the project went through a lengthy permitting process and approvals were received from all relevant agencies.

Title could have more impact - the results indicate there were minimal animal collisions with the turbine which could be highlighted right at the front. Currently, have to read ¾ down the abstract to find out this info. I can understand that the authors might consider this overselling their results but they present a good dataset and understanding of the limitations to make more of a statement.

While we appreciate the suggestion and sentiment, we do not want to overstate the implications of this study. Our concern is that if we did this, readers who skim the title and abstract might take home the message that we have quantitatively evaluated collision risk. We have left the title unchanged.

Authors have done a good job describing how the data collection changed throughout the deployment – Figure 3 was very helpful in this regard.

Thank you!

The methods left me wondering how the 5 annotator events were collated into a final event log? What was the variability/range between annotators? Presentation of some of the methods/data

concerning this and discussion around challenges, things to consider could be a valuable addition.

Please see below (L250) as our response there is redundant with how we would respond to this (which included adding supplemental material).

The presentation of Table 2, Fig 4, and Fig 5 could be more impactful. Currently, describes the table/figures and is a missed opportunity to present some of the trends around the environmental variables, e.g., most bird/seal events are at low flow speeds and high tidal range.

We have added a sentence to the initial presentation of these data indicating that trends are discussed in subsequent sections, and added references to the corresponding figures throughout the subsections on seals, fish, and seabirds.

Additional modifications based on reviewer-annotated pdf

Abstract
Strike “in this paper”

This has been removed from the abstract.

“Over fifty times more fish” → Suggest putting the number in parentheses

The text has been edited to include a parenthetical: (224 individual fish and 5 fish schools)

L77 - What does meso- macro- micro- scale mean in this context?

We agree that this language was non-specific and have revised the text according to Reviewer 1’s suggestion:

“Further, most studies to date have studied animal avoidance at larger scales, but there have been few reports of fine scale evasion behavior in the immediate vicinity of a tidal turbine.”

L112 - Suggest including this detail [on permitting] in the ethics statement

This is a valid point. We have modified the ethics statement to note that the project went through a lengthy permitting process and approvals were received from all relevant agencies.

Figure 2: My preference is to have a big picture location inset map for site maps. This map assumes your reader knows where Seattle is within US geography and based on the literature cited in the intro this work would have a much broader reach than the US.

It is not clear to me how b lines up with a? b represents the inlet to Sequim Bay which is presumably a smaller area than the white box in a but there does not seem to be any point in the box that is as narrow as the inlet in b? If it was possible to link up b within a more 1:1 might assist even if it's a point marker and can label Sequim Bay.

We initially chose not to include the full US because the area is so small relative to the US. In response to this suggestion we made a new figure (smaller version inserted here) that highlights the location in the context of a map of the US and parts of Mexico and Canada. A red dot is centered on the deployment location. Insets a and b now provide the bathymetry of Sequim Bay and the inlet. We believe this is more useful contextual information and appreciate the recommendation.

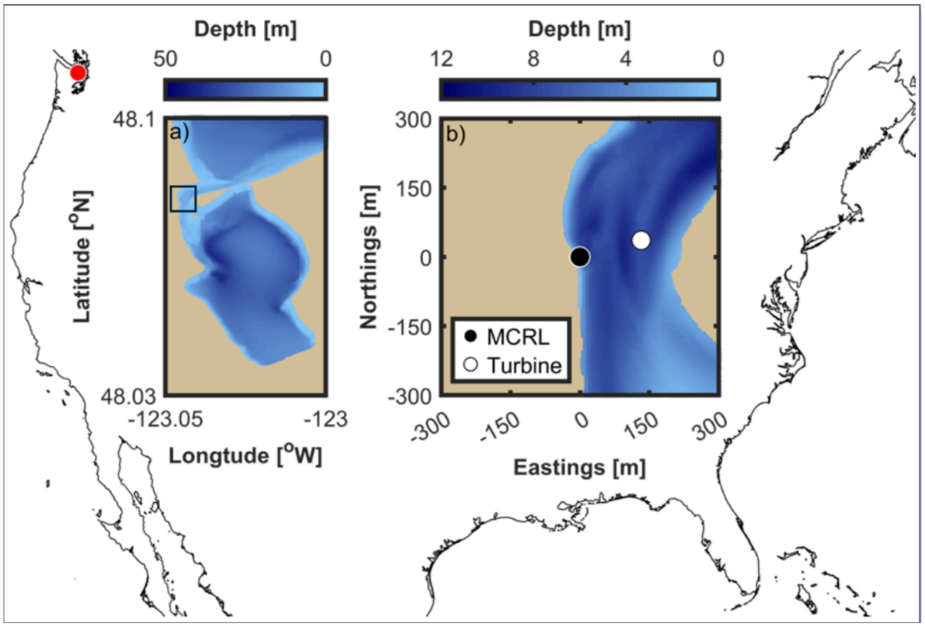

L190 - Strike “in this work”

This has been removed from the text.

L211 - Could add these maintenance/no date events on the fig 3 timeline with a *.

This is a good suggestion, and the figure has been updated accordingly. We also indicated on the figure the time window when the turbine was no longer operating (after February 11).

L219 - Presumably these multiple observations were only counted once but both datasets were used to interpret interactions

This is correct. All of the events identified are unique, even if they appear in two subsets. This is discussed in the Methods (L230) and has now been reiterated in the caption of Table 2.

In addition, we have inserted a clarifying comment on L266 which states:

“Events appearing in more than one data subset were consolidated such that they are represented in each subset in which they appeared, but the aggregated events presented in the Results section count reflect only unique events.”

L250 - Good number of annotators - any inter-annotator comparisons done? How were discrepancies between annotator quality controlled to produce the final list of annotations that was reviewed in further detail?

Yes, there were more annotators that we had intended to use in the project. This emerged for two reasons. First, over time we increased the scope but the initial annotators were no longer with our organizations (the first two were summer interns). Second, with available support we were able to extend the scope of our analysis. With that in mind, the supplemental material now contains considerably more details about how this was performed. This is too much information to include here, but the following image summarizes the workflow and is included in this new information.

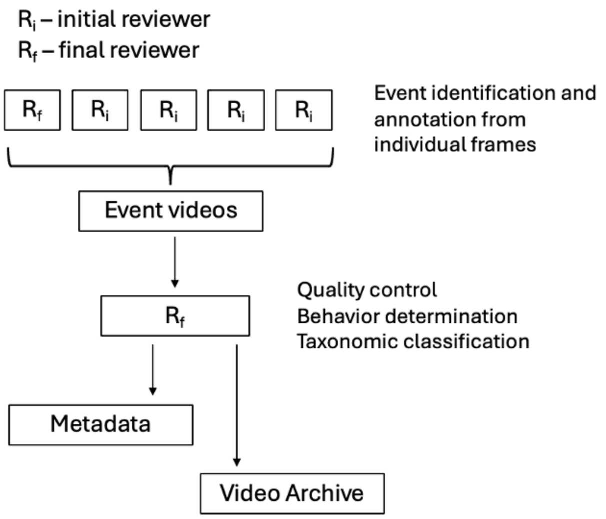

The initial reviewers, at the beginning of the process, were shown examples of common target types and also reviewed a subset of data with the final reviewer. They were then encouraged to identify any frame containing either a clear target or an ambiguous target. Thus, the data set produced by the initial reviewers contained far more potential targets than are included in our final dataset. It was the job of the final reviewer to pair these observations back to consistent observations. Birds, large fish, and seals were all generally clear and difficult to miss. Potential small fish were ultimately identified in high numbers by all of the initial reviewers. The final reviewer made a final decision on each event, and ultimately reviews hundreds (if not

thousands) of additional potential events were rejected. We figured this was the only way to consistently reconcile the contributions of multiple people.

Note also in several cases we did have initial reviewers revisit subsets of data previously processed by other initial reviewers. In these cases no additional targets of interest were identified.

We cannot say with certainty that small fish were counted at the same rate by all initial reviewers. However, as noted herein, we believe our observations likely considerably undercount those targets due to detection challenges and ambiguity. In light of all the factors, we feel good about the results. As a final note, if doing this again, we would want to ensure that we had sufficient support and time to use fewer reviewers to avoid the challenges herein.

Table S1 - Would love it if the table also included invertebrates

Because invertebrates were so frequently observed, and were not of regulatory concern, we did not typically annotate them during data review. However, the observed invertebrates were primarily kelp crabs (*Pugiettia producta*), which regularly climbed on the rotor while it was stationary, and jellyfish (unknown spp. & *Aurelia aurita*).

L208 - We have a description of what each Figure is but what are they contributing to the understanding of animal interactions with turbines? If they are not central to the overall narrative, recommend moving to supplemental but there seem to be some obvious takeaways worth including. e.g., Table 2 why less birds in subset 1? its there a seasonal components to the seabirds? fig 4 counts increase at high tide etc.

We have added explicit references to these figures throughout the results section. We also added more description of the timeseries figure (Figure 5) to the text. The original text said:

> "A representative time series of events from 2-8 February 2024 is shown in Fig 5, and a full list of all annotated events, from all three data subsets, can be found in S2 Event Data"

Beginning at line L321 it now states:

> "A representative time series of events from 2-8 February 2024 is shown in Fig 5. Among the patterns present in this time series are that bird detections were most common at high tides during daylight hours and that fish and seals were observed under a broad range of conditions. These trends are discussed in the subsequent sections and a full list of all annotated events, from all three data subsets, can be found in S2 Event Data."

L310 - Is this from the single annotator who did the species ID?

Yes, all reported results are from the final reviewer who did species ID. This has been clarified in the methods:

> "After initial annotation, each event was analyzed in detail by a single reviewer. Taxonomic classification was performed to the lowest level possible for each animal, and qualitative observations of animal behavior were logged. *These annotations were*

considered the final dataset that are discussed in the Results section and included in Supplemental Material." (The italics show the text that was added).

L310 - Reiterate overlap in detections between data subsets in the caption of Table 2

We agree this is a valuable thing to reiterate and have added the following to the caption. "Note that some events contained in data subset 2 are also contained in data subset 3."

Table 2 - Recommend putting the number of hours recorded or similar to put some context around these numbers - make it easy for the reader and put together vs flipping back to table 1.

The total number of hours recorded in each data subset has been added to the heading for each section of the table (i.e., Subset 1 (81.5 hrs recorded)).

L 336 - Should mention this in the methods

A statement regarding the chord length has been added to the methods. This statement, which was modified to address a comment from the other reviewer, now states:

"The four-bladed rotor is 1.19 m tall and 0.85 m in diameter and the chord length of the blades is 10.2 cm."

L369 - Cite table 2.

This reference has been added.

L402 - Figure 4 supports this, suggest citing here with the percentage

This reference has been added.

L415 - prey (a fish) → fish prey?

We agree this is more concise; the text has been revised accordingly.

General: Comments on figure placement

We formatted this submission using the PlosONE latex template. We will ensure that all figures are appropriately placed in the proofs of the final publication.

Other changes to the manuscript:

During our revisions, we made several small changes to the manuscript that were not in direct response to any reviewer comments. We have included a redline version of the manuscript and the most significant changes are described below.

- Caption 6 included a statement related to when the rotor was “moving.” The word “moving” was changed to “rotating.”
- L369 of revised manuscript previously stated:
  “An example of this interaction is shown in Fig 7.”

  It was modified to
  “An example fish collision is shown in Fig 7.”
- In the fish school section, we discussed that five schools evaded the rotor but felt we were missing a clarifying point. Specifically, schools were typically too dense to readily count individuals. Thus, the total number of encounters (and cases of avoidance) are not accounted for. We added the following (now at L402) to reflect this:

  “The individual fish associated with fish schools that evaded the rotor could not be readily counted and are therefore not included in the evasion/collision metrics presented for individual fish.”
  Since we cannot be quantitative we do not make an attempt, but the number of fish evasion is schools alone would significantly increase the total number of observations, thereby reducing the collision rate with respect to “encounters”
